# The Care of Hospital Floors

**Published:** 1911-08-26

**Authors:** 


### Hospital Architecture and Construction.

[Communications on this subject should be marked "Architecture" in the left-hand top corner of the envelope.]

### THE CARE OF HOSPITAL FLOORS.

### MATERIALS, POLISHES, AND POLISHING BY CONTRACT.

The floors of hospitals may be divided into four classes, viz. (1) marble; (2) cement; (3) wood; (4) patent floors, including linoleum.

Of marble floors, the usual form in sanitary blocks, operating theatres, etc., is terrazzo, or marble concrete; it is made up of irregular pieces of marble of varying size, with surface measurement of from $\frac{1}{4}$ inch to $\frac{5}{8}$ inch, laid to a depth of about $\frac{1}{2}$ inch in Portland cement on a mastic bed. It has the advantage of being laid *in situ*, and, therefore, it is easy to arrange a circular base to meet the wall finish and channels to take off water after cleaning down. The finish of this floor to bring it to an even surface is done by a soft sandstone wedged into a long handle, a clumsy-looking tool, but wielded by the Italians, who do most of the London work, with amazing dexterity. The stone is pushed and drawn back over the surface, which is well wetted, until a level and even face is obtained. Sometimes irregularities appear in the floor, and these, if they appear within the "making good" period of the contract, should be again worked down level by the builder; but if the foundation of the floor is solid and substantial no bad effects should develop. In this type of flooring cracks sometimes appear, which are due to settlement of the supporting walls. The only remedy is to have them cut out and made good. For cleaning such floors a powder is used, rubbed on with a damp cloth. "Gospo" is a well-known brand.

Cement floors also develop cracks due to settlements in the building, and sometimes bubbles appear, owing to careless mixing of materials. The great secret of a successful cement floor is hard trowelling by the workman in laying it, and a well-trowelled floor has a surface like steel. If not well trowelled a cement floor is always giving off a fine dust.

Wood floors in the form of blocks are still much in use. These are usually about 9 inches by 3 inches by $1\frac{1}{2}$ inch thick, grooved and tongued, and bedded in mastic. Wood is particularly sensitive to the atmosphere, and it can readily be imagined that considerable movement must take place in each block. When this individual movement is summed up over an area such as a ward floor would present, it will be understood that no amount of foresight will avoid an occasional apparent multiplicity of wide joints or a bulging up of several blocks. As each block is bedded in mastic and held to its neighbour by the union joint, the floor should spread or contract equally all over. To ensure this in domestic work, it is usual to allow a free space around the wall covered by the skirting. In hospital work, where we have usually the cavetto instead of a skirting, this space is lost owing to the desire to diminish the number of interstices. Another important point in block floors is the unequal amount of traffic they have to stand. A parallel may be found now in a London street (Waterloo Bridge Road). This is laid with jarrah, or some such wood blocks, close on 6 inches thick, and yet the joint lines which one would expect to find running at right angles to the direction of the road are found to be two irregular segments of circles, as if the heavy traffic in each direction—one of which is uphill, too—carries the road with it. Theoretically, this must have its parallel in the ward floor; certain parts have traffic, wheel and foot, while others under the beds are scarcely trodden. It would almost seem that a straight joint at the bed line would minimise the tendency to bulge.

Wood floors are generally treated with paraffin-wax forced into the pores of the wood and then polished with a mixture of beeswax and turpentine.

Hard scrubbing of floors thus treated is not recommended, either in the interests of the patients or of the floor, which may, however, lightly be sponged over before repolishing. Beeswax and turpentine gives a hard antiseptic surface.

Patent floors, including linoleum, vary in their constituents. A complete analysis of one type was given in this journal in the beginning of the year, but asbestos and sawdust are the chief materials, and they come under the same heading as wood floors as regards care. Probably no type of floor is so given to bulging unless expansion space is provided; for instance, by open lines round the beds as suggested in the case of wood blocks.

Owing to the popularity of the contract system, the problem of the polishing of the ward floor is a simpler one for hospital managers, or, rather, matrons, than it was some years ago. The firm of "Ronuk," for example, has arranged a system whereby for a fee depending upon the size of the surface needing polishing, and also the time of day at which this polishing has to be done, the wood floors can be scoured at intervals of four weeks. In between these regular polishings the ward maids do what sweeping and polishing is necessary, and their action is worth comparison with the "Ronuk" workers for this reason: The latter are men, and whether muscular action in the arms is the reason, or merely expert training, men are far superior to women for this purpose. They work more quickly and with better results.

As regards the fee charged, it must be remembered that hospitals are not the only institutions which are glad to have their polishing over and done with in the relatively small hours of day. The National Gallery is typical of the public institutions for which early polishing is not merely a convenience but a necessity. Competition for the services of the polishers is, therefore, very general, and before ten is a more expensive time in which to hire them than later.

